# Association of Myopic Deformation of Optic Disc with Visual Field Progression in Paired Eyes with Open-Angle Glaucoma

**DOI:** 10.1371/journal.pone.0170733

**Published:** 2017-01-23

**Authors:** Yu Sawada, Masanori Hangai, Makoto Ishikawa, Takeshi Yoshitomi

**Affiliations:** 1 Department of Ophthalmology, Akita University Graduate School of Medicine, Akita, Japan; 2 Department of Ophthalmology, Saitama Medical University, Saitama, Japan; Bascom Palmer Eye Institute, UNITED STATES

## Abstract

**Purpose:**

The influence of myopia on glaucoma progression remains unknown, possibly because of the multifactorial nature of glaucoma and difficulty in assessing a solo contribution of myopia. The purpose of this study is to investigate the association of myopia with visual field (VF) progression in glaucoma using a paired-eye design to minimize the influence of confounding systemic factors that are diverse among individuals.

**Methods:**

This retrospective study evaluated 144 eyes of 72 subjects with open-angle glaucoma, with similar intra-ocular pressure between paired eyes, spherical equivalent (SE) ≤ -2 diopter (D), and axial length ≥ 24 mm. Paired eyes with faster and slower VF progression were grouped separately, according to the global VF progression rate assessed by automated pointwise linear regression analysis. The SE, axial length, tilt ratio and torsion angle of optic discs, Bruch’s membrane (BM) opening area, and gamma zone parapapillary atrophy (PPA) width were compared between the two groups. Factors associated with faster VF progression were determined by logistic regression analysis.

**Results:**

The mean follow-up duration was 8.9 ± 4.4 years. The mean value of SE and axial length were -6.31 ± 1.88 D and 26.05 ± 1.12 mm, respectively. The mean global visual field progression rate was -0.32 ± 0.38 dB/y. Tilt ratio, BM opening area, and gamma zone PPA width were significantly greater in the eyes with faster VF progression than those with slower progression. In multivariate analysis, these factors were significantly associated with faster VF progression (all *P* < 0.05), while SE and axial length were not associated with it.

**Conclusion:**

In myopic glaucoma subjects, tilt of the optic disc and temporal shifting and enlargement of the BM opening were associated with faster rate of VF progression between paired eyes. This suggests that myopia influences VF progression in glaucomatous eyes via optic disc deformations rather than via refractive error itself.

## Introduction

The association of myopia with glaucoma is a focus of interest in the field of glaucoma. Previous studies have reported a high incidence of myopia in patients with glaucoma [1, 2]. A systematic review and meta-analysis of 13 studies, including 11 population-based studies, reported that the odds ratios of association between myopia and glaucoma were 1.88 for any myopic condition and 1.77 for low myopia (up to -3 diopter [D] in spherical equivalent [SE]) [2]. The authors concluded that myopia was a risk factor for the development of glaucoma. Despite its evident association with the development of glaucoma, the association of myopia with the progression of glaucoma remains controversial. Chihara et al. reported that severe myopia was a risk factor for visual field (VF) progression in patients with open-angle glaucoma (OAG) [3]. However, several other studies were unable to establish similar association [4–8]. An evidence-based review of 85 articles reporting risk factors for glaucomatous VF progression found myopia to be an unlikely risk factor [8].

Previous studies that reported the insignificant association of myopia and glaucoma progression used refractive error or axial length as an indicator of severity of myopia [4–6, 8]. Although refractive error and axial length are gold standards for describing the severity of myopia, they do not exactly indicate how optic discs are affected by the elongation of the globe in myopic eyes. Glaucomatous axonal damage is considered to occur primarily in the lamina cribrosa, which is a structural component of the optic disc [9, 10]. Therefore, we hypothesized that myopic deformations of optic discs rather than refractive errors might be more directly associated with glaucoma progression.

The inconsistencies in the reported association between myopia and glaucoma progression in previous studies may also be attributed to the difficulty in assessing the influence of myopia on glaucoma progression. Glaucoma is perceived as a multifactorial disease, involving many systemic factors, such as sex [11], age [4, 12], heredity [13], vascular conditions [14, 15], cerebrospinal fluid pressure [16], and diabetes [17]. Therefore, it is not easy to exclusively evaluate the effect of myopia on glaucoma progression. To minimize the influence of known and unknown confounding factors, which vary among individuals, paired-eye comparison has been effectively used in clinical studies [18–20]. Two eyes of the same individual are similarly influenced by systemic factors; therefore, paired-eye comparison allows eliminating the effects of confounding systemic factors. This allows us to hypothesize that, in cases where the rate of glaucoma progression varies between paired eyes with varying extents of myopia, any difference in glaucoma progression may be attributed to difference in extent of myopia between the eyes.

The purpose of the present study was to perform paired-eye comparisons to determine whether myopia was associated with glaucomatous VF progression. In addition to refractive error and axial length, we also evaluated factors that are related to the myopic deformation of optic discs.

## Materials and Methods

This retrospective study was approved by the institutional review and ethics boards of the Akita University Graduate School of Medicine, Akita, Japan, and followed the tenets of the Declaration of Helsinki. Participants did not provide written or vertical consent owing to the retrospective nature of this study.

### Study Subjects

We enrolled consecutive patients evaluated at the glaucoma clinic of the Akita University Graduate School of Medicine between May 2014 and April 2016. Their medical records were reviewed for data regarding the following: refraction test, best-corrected visual acuity, central corneal thickness (CCT) and axial length measured by ultrasound pachymetry (Tomey Corporation, Nagoya, Japan), Goldmann applanation tonometry, slit-lamp biomicroscopy, gonioscopy, dilated fundus stereoscopic examination, color fundus stereo photography (Canon, Tokyo, Japan), spectral domain-optical coherence tomography (SD-OCT; Spectralis, Heidelberg Engineering GmbH, Heidelberg, Germany), and standard automated perimetry (Humphrey Field Analyzer II 750; 24–2 Swedish interactive threshold algorithm; Carl Zeiss Meditec, Dublin, CA, USA).

We determined the baseline untreated intraocular pressure (IOP) as the average of at least two IOP measurements prior to the use of IOP-lowering medications, mean follow-up IOP as the average of all follow-up IOP measurements, and IOP fluctuation as the mean of standard deviations of all follow-up IOP measurements. The rate of IOP reduction was calculated as the percentage of difference between the baseline and mean follow-up IOP values. All patients underwent medical therapy during the follow-up period. In case of patients who underwent intraocular surgery or laser surgery, we only used the data recorded before surgery for analysis.

The inclusion criteria were as follows: (1) patients with OAG, with an open iridocorneal angle, glaucomatous optic disc changes such as localized or diffuse rim thinning and retinal nerve fiber defects, and glaucomatous VF defects corresponding to the glaucomatous structural changes. Glaucomatous VF defects were defined by glaucoma hemifield test results outside normal limits or the presence of at least three contiguous non-edge test points within the same hemifield on the pattern deviation plot at < 5%, with at least one of these points at < 1%, confirmed by two consecutive reliable tests (fixation loss rate, ≤ 20%; false-positive and false-negative error rates, ≤ 15%); (2) axial length ≥ 24.0mm and SE ≤ -2 D; If the eye was pseudophakic, pre-operative SE was used to evaluate the eligibility. (3) follow-up duration of at least 2 years, with at least five reliable Humphrey VF test results; (4) IOP of paired eyes as close to identical as possible, in order to eliminate the effect of IOP on VF progression. To ensure this, the difference in IOP between paired eyes in all baseline, mean follow-up, and fluctuation measurement was set at ≤ 1 mmHg in normal tension glaucoma (NTG) and ≤ 2mmHg in primary open-angle glaucoma (POAG) [21]; (5) similar CCT values of paired eyes, in order to eliminate the effect of CCT on VF progression. To ensure this, the difference in CCT between paired eyes was set at ≤ 10 μm; and (6) corrected visual acuity ≥ 20/30, in order to minimize the effect of media opacity. Both eyes of each subject had to satisfy these criteria for inclusion in the paired-eye study.

We excluded potential subjects according to the following criteria: (1) Subjects who had similar rates of VF progression in both eyes (difference of VF progression rate between paired eyes ≤ 0.1dB/year). These were excluded because paired eyes could not be distinguished as those with faster or slower progression; (2) Eyes with ocular injury or intraocular disease other than glaucoma; (3) congenital optic disc abnormalities; (4) eyes with extremely high myopia (axial length > 28.5mm or SE < -10D). These were excluded because of the difficulty in identifying the Bruch’s membrane (BM) termination in SD-OCT images as well as the increased risk of myopic macular changes that could affect the VF; and (5) eyes with mean deviation (MD) worse than -18.0 dB at the initial VF examination, in order to avoid the floor effect.

### Measurement of the Optic Disc Tilt and Torsion

Optic disc tilt and torsion were measured according to previously described criteria [22–25]. Tilt ratio was defined as the ratio between the longest and shortest diameters of the optic disc [22–24]. Optic disc torsion was defined as the deviation of the long axis of the optic disc from the vertical meridian [24, 25]. The vertical meridian was identified as a vertical line 90° from a horizontal line connecting the fovea and the center of the optic disc. The angle between the long axis of the optic disc and vertical meridian was defined as the torsion angle. A positive torsion value indicated inferior torsion, and a negative value indicated superior torsion. The absolute values of the torsion angle were used in the analysis to avoid compensation of the positive and negative torsion values.

### Assessment of the Bruch’s Membrane Opening Area

We assessed the BM opening area in infrared fundus images visualized on the display window of the Spectralis viewer. The details of this method were described previously [20, 26–29]. In brief, radial scan enhanced depth imaging SD-OCT was performed from the center of the optic disc and included 48 B-scan images, 3.8°apart, in each eye. Each B-scan image was constructed by averaging 42 frames. The termination of the BM was identified in 12 equidistant radial scans, and both sides of the termination were plotted on the scan line. The BM terminations plotted on the scan lines were manually delineated, and their extent was defined as the BM opening area. Magnification error was corrected according to the formula provided by the manufacturer, based on the results of autorefraction keratometry and focus setting during image acquisition. In cases where the image quality of the radial B-scan used for identifying BM termination was suboptimal, a neighboring image was used instead. In cases where the quality of both neighboring images was suboptimal, the eye was excluded.

**Fig 1:**
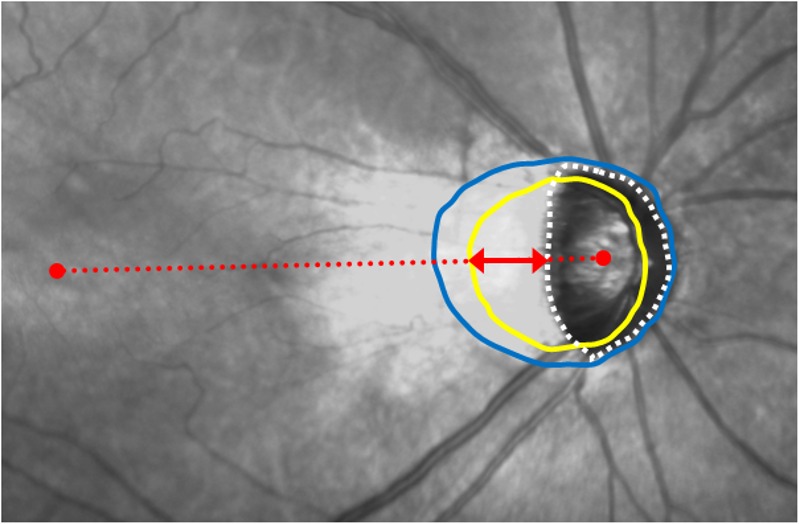
Burch’s membrane opening area and width of gamma zone parapapillary atrophy. The BM opening is shown as a yellow line and the optic disc margin as a white dotted line. The distance between the BM opening and temporal optic disc margin (red arrow) along the line connecting the fovea and the center of the optic disc (red dotted line) was defined as the width of the temporal PPA without BM (i.e. gamma zone). The blue line delineates the beta zone PPA margin.

The BM opening often shifted over the optic disc in the temporal direction, and its nasal part slid into the nasal region of the optic disc (Fig 1). The width of the temporal parapapillary atrophy (PPA) without BM (i.e. gamma zone PPA) was considered to present temporal shifting of the BM opening. It was measured along the line connecting the fovea and the center of the optic disc (Fig 1).

### Analysis of Visual Field Progression

We performed automated pointwise linear regression analysis using the Progressor software (Version 3.3, Medisoft, LTD, Leeds, UK). Details of the software have been described in previous studies [4, 30]. In brief, significant progressive VF test points were defined as those exhibiting progression rates < -1.0 dB/year for the inner points, and < -2.0 dB/year for the edge points, both with *P* < 0.01. The global VF progression rate of each eye was calculated by averaging the progression rates of all test points measured during the follow-up period. The number of the progressing points in each eye was determined. We divided the paired eyes of each subject into the faster and slower VF progression groups according to the global VF progression rate, and compared the SE, axial length, and parameters representing myopic deformation of optic discs, including tilt ratio, torsion angle, BM opening area, and width of gamma zone PPA between the two.

### Data Analysis

Comparison of variables between paired eyes was performed using the paired-sample *t* test. Conditional logistic regression analysis, which takes into consideration the correlation between paired eyes, was performed for the evaluation of factors associated with faster VF progression. Multivariate analysis was carried out with factors with *P* < 0.1 in the univariate analysis, using a backward elimination approach. Tilt ratio, BM opening area, and gamma zone PPA width were included in separate models because of the multicollinearity among them. Inter-observer reproducibility of the tilt ratio, torsion angle, and BM opening area measurements was assessed by determining the intraclass correlation coefficients (ICCs) of each variable, along with the corresponding 95% confidence intervals (CIs); 30 randomly selected eyes were assessed by two independent observers (YS and TY), and the ICC was calculated. Statistical analyses were performed using the SPBS ver. 9.66 [31]. Conditional logistic regression analysis was performed using the R software ver.3.1.1 (https://www.r-project.org/). The level of significance was set at *P <* 0.05, and all *P*-values were two-sided.

## Results

Among the 146 subjects with 292 eyes that were initially screened, we excluded the following (some for multiple reasons): difference in IOP between paired eyes > 1mmHg in NTG, or > 2 mmHg in POAG (n = 68); difference in CCT between paired eyes > 10μm (n = 10); OCT images of poor quality (n = 7); unreliable VF test results (n = 7); congenital optic nerve abnormalities (n = 3); and epiretinal membrane (n = 2). The remaining 144 eyes (72 subjects) were included in the analysis. The subjects included in the present study were all Japanese. Among included, 18 eyes of 10 subjects were pseudophakic. The ICCs for the measurement of tilt ratio, torsion angle, and BM opening area were 0.94 (95% CI, 0.91–0.97), 0.93 (95% CI, 0.90–0.96), and 0.96 (95% CI, 0.93–0.98), respectively.

The mean age of the included subjects was 56.2 ± 13.3 years, and the mean follow-up period was 8.9 ± 4.4 years (Table 1). The mean SE was -6.31 ± 1.88D; mean axial length, 26.05 ± 1.12mm; mean global VF progression rate, -0.32 ± 0.38 dB/year; and mean MD at initial examination, -6.60 ± 6.82 dB.

**Table 1:** Subject Demographics and Comparison between Paired Eyes with Faster and Slower Visual Field Progression.

	All Eyes	Paired Eyes with Faster and Slower VF Progression		
Factors	(n = 144)	Faster Progression (n = 72)	Slower Progression (n = 72)	*P* Value
Sex, n (male/female)	76/68			n/a
Age (yrs)	56.2±13.3			n/a
Follow-up period (yrs)	8.9±4.4			n/a
Number of VF tests	14.7±7.7			n/a
**Global VF progression rate (dB/y)**	-0.32±0.38	**-0.43±0.44**	**-0.20±0.25**	**<0.0001**
**Number of progressing points**	2.9±3.9	**4.2±4.5**	**1.6±2.8**	**<0.0001**
**MD—Initial examination (dB)**	-6.61±6.82	**-9.13±7.61**	**-4.09±4.86**	**<0.0001**
**MD—Last examination (dB)**	-9.24±7.50	**-12.48±7.66**	**-5.99±5.83**	**<0.0001**
Spherical equivalent (diopter)	-6.31±1.88	-6.43±1.82	-6.19±1.94	0.1607
Axial length (mm)	26.04±1.12	26.10±1.17	25.99±1.08	0.0962
Central corneal thickness (μm)	522.4±29.5	522.5±29.9	522.2±29.5	0.6094
IOP Untreated (mmHg)	18.5±3.6	18.6±3.7	18.5±3.6	0.1957
Follow-up mean (mmHg)	14.4±2.0	14.4±2.1	14.4±2.0	0.6901
fluctuation (mmHg)	1.76±0.57	1.77±0.58	1.75±0.56	0.3125
Reduction rate (%)	21.1±11.7	21.3±11.8	21.0±11.8	0.2495
**Tilt ratio**	1.30±0.30	**1.36±0.34**	**1.25±0.23**	**<0.0001**
Torsion angle (degree)	6.3±5.1	6.5±5.0	6.0±5.4	0.4737
**BM opening area (mm**^**2**^**)**	2.47±0.61	**2.60±0.66**	**2.34±0.52**	**<0.0001**
**Gamma zone PPA width (μm)**	294.5±285.0	**344.7±306.2**	**244.4±256.7**	**<0.0001**

Values are shown as means ± standard deviations. Comparisons were performed using paired-sample *t* test. Statistically significant values are shown in bold.

VF = visual field; MD = mean deviation; IOP = intra ocular pressure; BM = Bruch’s membrane; PPA = parapapillary atrophy; n/a = not applicable.

In comparison between paired eyes with faster and slower VF progression, global VF progression rate was -0.43±0.44 dB/y vs. -0.20±0.25 dB/y (*P* < 0.0001; Table 1). SE and axial length were not different between groups. Tilt ratio, BM opening area, and gamma zone PPA width were greater in the eyes with faster progression (all, *P* < 0.0001). Torsion angle was not different between groups.

Upon univariate conditional logistic regression analysis, the MD at initial examination, tilt ratio, BM opening area, and gamma zone PPA width were found to be associated with faster VF progression (all, *P* < 0.01; Table 2), while SE and axial length were not significantly associated with it. Upon multivariate analysis, the MD at initial examination, tilt ratio, BM opening area, and gamma zone PPA width retained their significance of association (all, *P* < 0.05; Table 2).

**Table 2:** Factors Associated with the Faster Visual Field Progression.

	Univariate Analysis			Multivariate Analysis 1^a^		
Factors	Odds Ratio	95% CI	*P* Value	Odds Ratio	95% CI	*P* Value
**MD at initial examination**	**0.72**	**0.61–0.84**	**<0.0001**	**0.74**	**0.63–0.88**	**0.0005**
Spherical equivalent	0.81	0.55–1.17	0.1800			
Axial length, per 0.1mm increase	1.07	0.96–1.20	0.1200			
**Tilt ratio, per 0.01 increase**	**1.08**	**1.03–1.12**	**0.0008**	**1.05**	**1.01–1.05**	**0.0295**
Torsion angle	1.04	0.97–1.14	0.2200			
**BMO area, per 0.1mm**^**2**^ **increase**	**1.38**	**1.16–1.63**	**0.0003**			
**Gamma zone PPA width**	**1.01**	**1.00–1.01**	**0.0014**			
	Multivariate Analysis 2^b^			Multivariate Analysis 3^c^		
	Odds Ratio	95% CI	*P* Value	Odds Ratio	95% CI	*P* Value
**MD at initial examination**	**0.71**	**0.59–0.85**	**0.0002**	**0.72**	**0.61–0.86**	**0.0001**
Spherical equivalent						
Axial length, per 0.1mm increase						
**Tilt ratio, per 0.01 increase**						
Torsion angle						
**BMO area, per 0.1mm**^**2**^ **increase**	**1.34**	**1.13–1.61**	**0.0019**			
**Gamma zone PPA width**				**1.01**	**1.00–1.11**	**0.0238**

Statistical analysis was performed using conditional logistic regression analysis. Statistically significant values are shown in bold. Factors with *P* < 0.10 in the univariate analysis were included in the multivariate analysis.

The multivariate analysis was performed separately using tilt ratio, BMO area, and gamma zone PPA width because of the multicollinearity among them.

^a^ MD at initial examination and tilt ratio were included in the model.

^b^ MD at initial examination and BMO area were included in the model.

^c^ MD at initial examination and gamma zone PPA width were included in the model.

CI = confidence interval; MD = mean deviation; BMO = Bruch’s membrane opening; PPA = parapapillary atrophy.

**Fig 2:**
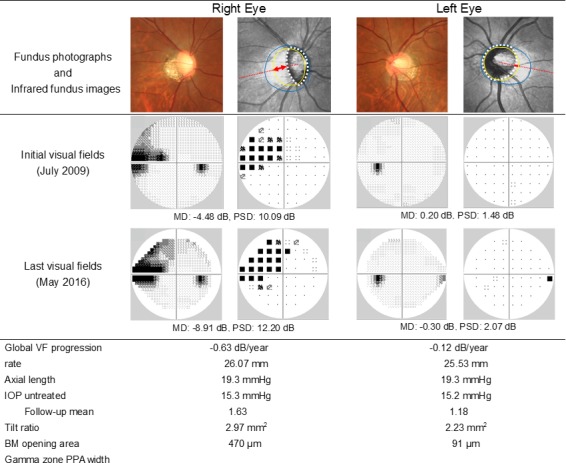
A representative case with a different amount of myopic deformation of the optic discs between paired eyes. Yellow lines in the infrared fundus images indicate BM openings, and white dotted lines delineate the optic disc margins. Red arrows indicate the width of gamma zone PPA along the red dotted lines connecting the fovea and the center of the optic disc. Blue lines delineate the beta zone PPA margins. Humphrey VF data are shown in gray scale and pattern deviation plot.

Fig 2 shows a representative case of a OAG patient with a different amount of myopic deformation of the optic discs between paired eyes.

In this 47-year-old female patient with OAG, the right eye, which showed faster VF progression, exhibited greater axial length, tilt ratio, BM opening area, and gamma zone PPA width than the left eye, which showed slower progression.

## Discussion

As far as we are aware, this study is the first to evaluate the association of myopia with VF progression in OAG eyes using a paired-eye study design to eliminate the effect of systemic factors that vary among individuals. In addition, we ensured that the IOP and CCT between paired eyes were similar, in order to minimize their effects on VF progression. In these study settings, as consistent with the results of previous studies, we found no association between myopic refractive errors and VF progression. However, we found that disc tilt accompanying temporal shifting and enlargement of BM opening was independently associated with faster VF progression. This suggests that myopia influences glaucomatous VF progression not via refractive error or axial length itself, but via myopic optic disc deformation.

The results of the present study are consistent with those of previous studies that found no significant association between refractive errors and glaucomatous VF progression [4–8]. In the Advanced Glaucoma Intervention Study, refractive error was not found to be one of the predictive factors for glaucomatous VF progression, which included age and IOP fluctuation [4]. More recently, the rate of VF progression was compared among myopic eyes with varying extents of refractive errors, and none of the levels of refractive errors were found to be associated with the rate of progression [5, 6]. In accordance with the results of the previous studies, our results confirmed that refractive error was not associated with VF progression even after eliminating the effects of systemic factors.

In the present study, we demonstrated an association between tilt ratio of the optic disc and faster VF progression. A previous study had reported the association of disc tilt with bihemispheric retinal nerve fiber layer defect (RNFLD) in early-stage glaucoma [32]. The authors referred to a finding of another study, in which bihemifield VF defects in early-stage glaucoma resulted in an increased risk of VF progression [30], and hypothesized that disc tilt could be a risk factor for faster VF progression. Their study assessed bihemispheric RNFLD, and did not actually address glaucoma progression. Our result is in line with theirs, and supports their hypothesis by demonstrating actual association between them.

A previous study reported a smaller probability of glaucoma progression in the eyes with tilted disc compared to those with non-tilted disc [7]. The result of the present study appears to be contradictive to the results of the previous study; however, the discrepancy might be explained by many differences between two studies, mostly in methodology. The previous study divided subjects into tilted and non-tilted disc groups according to the tilt ratio with a cut off value 1.3. However, since tilt ratio is a continuous value, it remains unclear if it is appropriate to determine a cut off value and divide subjects according to it. The present study used tilt ratio as a categorical value, and this method is more practical and accurate in evaluating the association of disc tilt and VF progression. In addition, the previous study chose one eye of the consecutive subjects to construct study groups. Systemic risk factors were not adjusted between them, thus, glaucoma progression was affected not only by myopia but also by other varying systemic factors. The present study employed paired eye setting, and it helped adjusting many known and unknown confounding factors between groups; therefore, the susceptibility was operationalized mostly by myopia. Also, in the previous study, mean follow-up IOP was significantly lower in the tilted disc group than in the non-tilted disc group, and baseline IOP was lower in the former with marginal significance. Since IOP plays a role in glaucoma progression [12, 32, 33], the lower IOP in the tilted disc group could cause slower progression in this group. The present study employed the strictest criteria of equal IOP between paired eyes previously reported [21] to minimize the effect of IOP.

The association of tilting of the optic disc and temporal shifting of the BM opening with faster VF progression might be explained by the increased susceptibility to the glaucomatous stress. Temporal shifting of the BM opening relative to the scleral canal opening occurs during the elongation of the globe along with the tilting of the optic disc. It creates a scleral region bared of BM on the temporal side of the optic disc. This region is called gamma zone PPA [28, 34]. In highly myopic eyes, markedly elongated and thinned sclera is observed in parapapillary region only with retinal nerve fiber layer but without BM and choroid overlying it [35]. According to the previous studies, the thickness and structural properties of parapapillary sclera are important to maintain normal biomechanics of lamina cribrosa [36–39]. Thinner parapapillary sclera is associated with higher tension in the lamina cribrosa at a given trans-laminar cribrosa pressure gradient. The absence of BM might influence on the stability of the architecture of the retina-choroid complex. Further, the elongation of the peripapillary sclera leads to the increased distance between the peripapillary arterial circle of Zinn-Haller and the optic disc border [40, 41]. Since the arterial circle of Zinn-Haller supports the blood vessels in the optic disc, particularly in lamina cribrosa [42], the increased distance may cause a malperfusion of lamina cribrosa. These mechanisms are hypothesized to explain increased glaucoma susceptibility in highly myopic eyes. Although the extent was smaller, the same morphological changes were observed in our subjects with moderate myopia, such as wide gamma zone without overlying BM and choroid. Therefore, we consider these hypotheses may also be applicable to explain the findings of our study.

The present study has some limitations. The first is its retrospective design. Prospective studies should be conducted to confirm whether eyes with greater myopic deformation of optic discs subsequently develop faster VF progression. The second is the possibility of subject selection bias. We employed strict inclusion criteria, including similar IOP and CCT values between paired eyes, to assess the exclusive influence of myopia. These criteria resulted in the exclusion of a certain proportion of potential subjects, thus decreasing the sample size. Further studies are required to examine whether our findings are applicable to larger populations. The third is a relatively small threshold difference in VF progression between faster and slower progression groups (> 0.1 dB/year). This threshold was set because of the small difference of the progression rate between groups (-0.43 dB/year in faster progression group vs. -0.20 dB/year in slower progression group). This small difference might owe to the paired-eye design of the study. With this study design, the difference of the rate of progression is essentially operationalized by the difference of myopia between paired eyes; however, two eyes of the same subject are not likely to have extremely different level of myopia. Relatively small difference of the level of myopia might lead to the small difference of the progression rate between groups. Further studies are needed to test the hypothesis in other study settings with greater threshold differences.

## Conclusions

Our paired-eye study demonstrated significant association between glaucomatous VF progression and myopic optic disc deformation such as disc tilt and temporal shifting and enlargement of the BM opening. These results suggest that myopia influences glaucomatous VF progression via optic disc deformations. In the management of patients with OAG with myopia, fellow eyes with greater optic disc deformations should be considered to be at a greater risk for faster VF progression.

## Supporting Information

S1 File Data of paired eyes with faster and slower visual field progression. Data of paired eyes with faster and slower visual field progression of each patient are presented.
(CSV)
